# Validation in trial-based economic evaluations: a scoping review protocol

**DOI:** 10.1136/bmjopen-2026-119447

**Published:** 2026-09-17

**Authors:** May Ee Png, Kamran Ahmad Khan, Tracey H Sach, Helen Parsons, Vivan Patel, Phuong Bich Tran, Dyfrig A Hughes, Nia Roberts, Rebecca Kandiyali

**Affiliations:** 1 Nuffield Department of Primary Care Health Sciences, University of Oxford, Oxford, UK; 2 Warwick Medical School, University of Warwick, Coventry, England, UK; 3 School of Primary Care, Population Sciences and Medical Education, University of Southampton, Southampton, UK; 4 Centre for Health Economics and Medicines Evaluation, Bangor University, Bangor, UK; 5 Bodleian Health Care Libraries, University of Oxford, Oxford, UK

**Keywords:** Review, HEALTH ECONOMICS, Methods

## Abstract

**Introduction:**

Economic evaluations conducted alongside clinical trials play a key role in informing healthcare decision-making. While structured validation frameworks are well established for model-based economic evaluations, there is no agreed approach to validating trial-based economic evaluations. Validation activities in this context are variably defined, inconsistently reported and often embedded implicitly within analytical practice. As part of a wider programme of work to develop a validation tool for trial-based economic evaluation, this scoping review aims to systematically map how validation is conceptualised, described and undertaken in the literature.

**Methods and analysis:**

This protocol describes a planned scoping review. The completed review will be conducted in accordance with the Preferred Reporting Items for Systematic Reviews and Meta-Analyses extension for Scoping Reviews guidelines. We will search MEDLINE, Embase and the International Network of Agencies for Health Technology Assessment (INAHTA) database. Grey literature will be identified through targeted searches of health technology assessment and guideline-producing organisation websites, with ProQuest searched for doctoral dissertations only and reference lists of included sources screened. We will include trial-based economic evaluations, methodological and conceptual papers, guidance documents and empirical studies that describe or assess validation activities relevant to trial-based economic evaluation. Title and abstract screening and full-text review will be undertaken independently by two reviewers. Data extraction will be conducted by one reviewer, with all extracted records independently verified by a second reviewer. Data will be extracted using a structured form capturing study characteristics and validation activities. Findings will be synthesised descriptively using tables and narrative summaries to map validation concepts and practices.

**Ethics and dissemination:**

Ethical approval is not required as this review will use published literature only. Findings will be disseminated through peer-reviewed publication, conference presentations and engagement with stakeholders, and will inform subsequent consensus-based work to develop a validation tool for trial-based economic evaluation.

**STRENGTHS AND LIMITATIONS OF THIS STUDY**

- The review will include both peer-reviewed literature and targeted grey literature from key health technology assessment and methodological organisations, improving coverage of guidance and practice.
- Consistent with scoping review methodology, the review will not assess the methodological quality of included studies or the effectiveness of validation approaches.
- Eligibility requires validation to be explicitly described in the abstract. Heterogeneous and inconsistent terminology across the literature may further contribute to the exclusion of studies where validation activities are undertaken but not foregrounded.
- The review excludes standalone validation studies of outcome measurement instruments or valuation methods unless explicitly framed within trial-based economic evaluation.

## Introduction

 Economic evaluations conducted alongside clinical trials are widely used to inform healthcare decision-making and considerations around the efficient use of healthcare resources.^1^ By combining cost and outcome data collected within trials, trial-based economic evaluations are often viewed as having high internal validity and close alignment with clinical evidence^2^ and are therefore widely used in health technology assessment submissions and applied research, particularly to inform model-based economic evaluations.^3^

Ensuring the validity of trial-based economic evaluations is critical, as errors or inappropriate assumptions in design, data collection, analysis or interpretation may directly affect the credibility of results and their use in decision-making. Validation in this context may encompass a range of activities, including checking analytical code, assessing internal consistency, scrutinising assumptions, comparing results with external data and considering face or conceptual validity.^4^ In methodological discourse, validation is closely related to concepts such as reliability, transparency and reproducibility. These terms are sometimes used interchangeably, but they reflect distinct dimensions of research integrity. Transparency and reproducibility concern the clarity and completeness with which methods and assumptions are reported and can be independently replicated, whereas validation focuses on assessing whether the economic evaluation appropriately represents underlying data, assumptions and analytical processes. However, such validation activities are frequently implicit, variably defined or poorly documented in published studies.

While structured validation frameworks are well established for model-based economic evaluations,^5–7^ there is no agreed approach to validating trial-based economic evaluations. Although concepts such as internal validity, external validity, generalisability and transferability are frequently discussed in relation to trial-based economic evaluations, they are not typically brought together within an overarching framework of validation. Consequently, it remains unclear which activities should be considered validation in this context and how they relate to existing concepts used in trial-based research. Model-based evaluations benefit from dedicated guidance and tools that distinguish different types of validity and recommend systematic validation processes, such as the assessment of internal consistency, external validity and predictive performance.^4 8^ In contrast, there is no established validation framework specially tailored to trial-based economic evaluations. Recent work has proposed a risk-proportionate framework for the validation of statistical programming within clinical trials, emphasising reproducibility and regulatory compliance.^9^ However, such guidance focuses on statistical programming processes and does not address the broader methodological and conceptual validation challenges specific to trial-based economic evaluations, including costing methods, valuation of outcomes and economic decision metrics.

The absence of a shared conceptualisation and structured approach to validation presents challenges for transparency, reproducibility and comparability across trial-based economic evaluations. Although reporting standards such as the Consolidated Health Economic Evaluation Reporting Standards (CHEERS) 2022 promote transparency in reporting economic evaluations, they were not designed as tools for systematic validation or error-checking.^10^ Similarly, the development of Health Economics Analysis Plans (HEAPs) has strengthened the prospective specification and transparency of trial-based economic analyses. However, HEAPs focus on defining analyses a priori rather than providing a structured framework for assessing the validity of methods, assumptions or results.^11^ As a result, important validation activities may remain undocumented or inconsistently applied.

As part of a wider programme of work to develop a validation tool for trial-based economic evaluations, this scoping review aims to systematically map how validation is conceptualised, described and undertaken in published and grey literature. Specifically, the review will identify the types of validation activities reported, the components of trial-based economic evaluations to which they are applied, the stages at which validation occurs, the methods and data sources used and how these activities are described and reported within published and grey literature. By providing an overview of existing concepts and practices, this review will inform subsequent consensus-based work to support best practice in the validation of trial-based economic evaluations.

## Methods and analysis

### Study design

This protocol describes a planned scoping review. The completed review will be reported in accordance with the Preferred Reporting Items for Systematic Reviews and Meta-Analyses extension for Scoping Reviews (PRISMA-ScR).^12^ In the absence of a dedicated reporting guideline for scoping review protocols, this protocol has been informed by the PRISMA-Protocols guideline (online supplemental table S1).^13^ A scoping review methodology was chosen because validation in trial-based economic evaluation is poorly defined and variably described, making it necessary to first map existing concepts and practices before more focused synthesis is possible.

### Eligibility criteria

For the purposes of this review, a trial-based economic evaluation is defined as a comparative analysis of costs alone or costs and health outcomes conducted alongside a clinical trial, including cost-effectiveness, cost–utility, cost–benefit and cost–consequence analyses. Both full and partial economic evaluations will be eligible, provided they are embedded within a clinical trial. Studies reporting only descriptive resource use or cost data without comparative economic analysis will be excluded.

We will include: (1) trial-based economic evaluations that explicitly describe validation activities and (2) methodological, conceptual or guidance papers that explicitly address validation in the context of trial-based economic evaluations.

We will include studies conducted in any health condition, patient population or setting, with no restrictions by disease area, population group, country or healthcare system.

Eligible sources that include an abstract must explicitly describe validation activities in relation to a trial-based economic evaluation within the abstract. This requirement reflects the aim of the review to map validation as an articulated construct rather than routine or implicit quality assurance practices. We acknowledge that some trial-based economic evaluations may undertake validation activities that are not explicitly reported in abstracts; such studies will not be captured by this approach. However, for grey literature sources that do not include an abstract, eligibility will be assessed at the full-text stage. In all cases, the focus of this review is on validation that is conceptually foregrounded and reported as a substantive methodological component.

Sources focusing on model-based economic evaluations will be assessed at full text during the scoping phase to inform understanding of validation concepts but will be excluded from data extraction and synthesis unless validation was explicitly described in relation to trial-based economic evaluation. Validation may include, but is not limited to, internal validity, external validity, face or content validity, cross-validation, conceptual validity, calibration, consistency or related approaches. These domains are informed by the definitions provided by the ISPOR–SMDM Modelling Good Research Practices Task Force-7 framework, and will guide initial classification during data extraction.^14^ Where validation activities fall outside these domains, additional categories will be developed inductively to reflect trial-specific considerations.

We will exclude studies that (1) do not relate to trial-based economic evaluation or (2) do not explicitly address validation of such evaluations. This includes studies focused solely on psychometric validation of quality-of-life instruments, as these concern validation of measurement instruments rather than validation of the economic evaluation itself. We will also exclude epidemiological or clinical models without an economic evaluation component; service delivery or intervention design frameworks that do not explicitly address validation of an associated trial-based economic evaluation; narrative commentaries or editorials with no empirical or methodological validation content; and validation of clinical trial processes or data collection methods not explicitly linked to trial-based economic evaluation.

### Information source

An initial search of electronic bibliographic databases including MEDLINE (OvidSP), Embase (OvidSP) and the International Network of Agencies for Health Technology Assessment (INAHTA https://database.inahta.org/) database was conducted from inception to 13 August 2025 (online supplemental table S2). The choice of databases was informed by published evidence on the identification of economic evaluations, which supports the use of key biomedical and health technology assessment databases.^15^ The search strategy will be updated if more than 12 months have elapsed between the date of the last search and completion of the review.

Grey literature was identified through targeted searches of health technology assessment agency and guideline-producing organisation websites, including the National Institute for Health and Care Excellence (NICE) and NICE Decision Support Unit, the Canadian Agency for Drugs and Technologies in Health (CADTH), the Pharmaceutical Benefits Advisory Committee (PBAC), the Medical Services Advisory Committee (MSAC), the Institute for Clinical and Economic Review (ICER), the WHO, the European Commission and the International Society for Pharmacoeconomics and Outcomes Research (ISPOR). Grey literature searches were conducted on 17 September 2025. ProQuest Dissertations & Theses (Global) was searched for doctoral dissertations only. A list of grey literature sources searched is provided in the online supplemental table S3.

In addition, reference lists of relevant systematic reviews identified through the searches were screened to identify further eligible sources.

### Search strategy

The search strategy was developed to identify literature relating to validation in economic evaluation, with the support of an information specialist (NR). The strategy combined terms relating to economic evaluation, trials and validation concepts, without restricting searches to trial-based economic evaluations at the search stage. Validated search strategies were used for the economic and trial elements of the search.^16–18^

Database-specific search strategies were adapted for MEDLINE, Embase and the INAHTA database. The full search strategies for all electronic databases, including the exact search terms and combinations used, are provided in online supplemental table S2.

For grey literature sources (online supplemental table S3), targeted keyword searches were undertaken using the root term “valid” to capture variations such as validity and validation. For doctoral dissertations, ProQuest was searched using a structured search strategy combining terms for economic evaluation and validation (title: “economic evaluation” OR cost-effectiveness OR cost-utility; and validation OR validate OR validity), with results limited using the ProQuest filter for doctoral dissertations.

### Selection of sources of evidence

Records identified from electronic database searches will be imported into Covidence, where duplicates will be removed automatically. Grey literature records will be managed and screened using Covidence. Titles and abstracts will be screened to assess potential relevance against the eligibility criteria. Full texts of potentially relevant sources will be retrieved and assessed for inclusion.

Title and abstract screening and full-text review will be conducted by two reviewers. One reviewer (MEP) will assess all records, with the remaining reviewers dividing the records between them. Disagreements will be resolved through discussion, with involvement of a third reviewer if necessary. Reasons for exclusion at the full-text stage will be recorded and reported.

### Data extraction

Data will be extracted from included sources using a bespoke structured data extraction form developed in Microsoft Excel for this review. The form will be designed to ensure consistent capture of information relevant to validation in trial-based economic evaluation and will be applied systematically across all included studies. Data extraction will be undertaken by a single reviewer (MEP). All extracted records will be independently verified for accuracy and consistency by a second reviewer, with verification responsibilities divided among members of the review team. Any discrepancies will be resolved through discussion within the review team.

The data extraction form will be piloted on a small number of included studies and refined as necessary to ensure clarity and consistency. Any uncertainties arising during data extraction will be discussed within the review team where required.

### Data items

Data items extracted from included sources will capture study characteristics and validation-related information relevant to trial-based economic evaluation. Extracted study characteristics will include study type, type of economic evaluation, health area and country of analysis.

Validation-related data items will include the focus of validation (eg, internal validity, external validity, face or content validity, cross-validation, predictive validity), the aspect being validated (e.g. resource use, costs, outcomes), and the stage of the economic evaluation at which validation occurs (design and recruitment, data collection or analysis). We will also extract information on the basis for validation (eg, use of internal trial data, external empirical data, logic or internal consistency checks, theory or conceptual frameworks, or expert judgement), data sources used where validation is empirical, and the methodological approach taken to undertake validation. Where reported, we will record whether validation was undertaken as part of a formal quality assurance process and the role of the individual(s) performing the validation (eg, internal team member, independent statistician or external reviewer).

In addition, we will extract information on how validation is described and operationalised, including the clarity with which the concept of validation is defined within each source. Reviewer notes will be used to record relevant contextual information, including quotations where helpful to clarify how validation is presented.

A detailed data extraction form, including all data items and response options, is provided in the online supplemental material table S4.

As this scoping review aims to comprehensively map how validation is conceptualised and operationalised in trial-based economic evaluation, no formal prioritisation of outcomes will be undertaken. All validation-related data items will contribute equally to the descriptive and conceptual synthesis.

### Critical appraisal of individual source

No formal critical appraisal or assessment of methodological quality will be undertaken. This scoping review aims to map how validation is conceptualised and undertaken in trial-based economic evaluation, rather than to evaluate the quality, effectiveness or appropriateness of individual validation approaches. Consistent with scoping review methodology, the purpose of this review is to identify the range and characteristics of existing evidence and to clarify key concepts, rather than to make judgements about study quality.

### Synthesis of results

Extracted data will be synthesised narratively to map how validation is conceptualised and undertaken in trial-based economic evaluation. Findings will be summarised using tables and narrative descriptions to characterise validation activities across studies, including the focus and object of validation, the stage of the economic evaluation at which validation occurs, and the basis, data sources and methods used.

The clarity with which validation is defined and operationalised will be summarised to highlight variation in how validation concepts are described across sources. No quantitative synthesis or meta-analysis will be undertaken.

### Patient and public involvement

Patient and public involvement (PPI) members will be involved in discussing the findings of the scoping review once the data have been collected and once early ideas have been developed. They will help us judge whether the findings are clear, relevant and easy to understand, and whether they are presented in a helpful way. PPI members will consider whether approaches used to assess trial-based economic evaluations reflect what patients and the public see as important, and whether these approaches are understandable and meaningful in practice.

## Ethics and dissemination

Ethical approval is not required for this study as it involves the review and synthesis of published and publicly available literature only and does not involve the collection of primary data from human participants or animals.

Findings from this scoping review will be disseminated through publication in a peer-reviewed journal and presentation at relevant academic and methodological conferences. Results will also be shared with methodological stakeholders with an interest in trial-based economic evaluation and will inform subsequent consensus-based work to support the development of a validation tool for trial-based economic evaluation. Key findings will be summarised in accessible formats and shared via the project webpage to facilitate engagement with PPI and engagement groups and the wider public.

## Supplementary material

10.1136/bmjopen-2026-119447 online supplemental file 1
