# Supplementary material for: Validation in trial-based economic evaluations: a scoping review protocol
Source: BMJ Open. 2026 Sep 17;16(9):e119447. doi: 10.1136/bmjopen-2026-119447 (PMC13599820; doi:10.1136/bmjopen-2026-119447)
Supplement: Supplementary file 1 [file bmjopen-2026-119447.reviewer_comments.pdf]

PEER REVIEW HISTORY

BMJ Open publishes all reviews undertaken for accepted manuscripts. Reviewers are asked to complete a checklist review form and are provided with free text boxes to elaborate on their assessment. These free text comments are reproduced below.

ARTICLE DETAILS

Title (Provisional)

Validation in trial-based economic evaluations: a scoping review protocol

Authors

Png, May Ee; Khan, Kamran Ahmad; Sach, Tracey H; Parsons, Helen; Patel, Vivan; Tran, Phuong Bich; Hughes, Dyfrig A; Roberts, Nia; Kandiyali, Rebecca

---

VERSION 1 - REVIEW

**Reviewer**	**1**
**Name**	**Humphreys, Ioan**
**Affiliation**	**Swansea University, Centre for Health Economics**
**Date**	**07-Jul-2026**
**COI**	**None**

A very timely, useful and welcome SR on trial-based economic evaluations.

**Reviewer**	**2**
**Name**	**Chattopadhyay, Sajal**
**Affiliation**	**Centers for Disease Control and Prevention, Community Guide**
**Date**	**27-Jul-2026**
**COI**	**None**

This is a well-written scoping review protocol that aims to systematically map how validation in trial-based economic evaluations is conceptualized, described, and undertaken in literature. I have a major concern about the statement that this scoping review will be conducted in accordance with the PRISMA-ScR guidelines. PRISMA-ScR is a reporting guideline for the final published scoping review, not technically a protocol template. There is no PRISMA-P equivalent specifically for scoping review template. For the protocol stage, the field instead leans on: 1) PRISMA-ScR checklist, applied prospectively — most protocols map their planned methods section onto the PRISMA-

ScR items in advance, so the eventual review naturally satisfies reporting requirements; and 2) JBI methodology for scoping reviews — the companion methodological guidance (distinct from the reporting checklist) that PRISMA-ScR itself was built alongside, covering how to actually design and conduct each step (Ref: Peters MDJ, Marnie C, Tricco AC, et al. Updated methodological guidance for the conduct of scoping reviews. JBI Evid Synth. 2020;18(10):2119-2126. doi:10.11124/JBIES-20-00167).

Also, the third paragraph of the Introduction raises an important conceptual distinction worth preserving clearly in the text: model-based economic evaluations (MBEEs) rely on an established, named validation taxonomy — face validity, internal verification, cross-validation, and external/predictive validation — drawn from Eddy et al. (2012) and operationalized in tools like AdViSHE. Trial-based economic evaluations (TBEEs), by contrast, rarely use "validation" as a term at all. Instead, the literature describes related but distinct concerns using different vocabulary: internal validity, external validity/generalizability, and transferability. This difference exists because models and trials rest on different epistemic foundations. A model has no single ground truth of its own — it synthesizes data from multiple sources, so its credibility must be checked against external benchmarks such as registries, comparator models, or longer-term follow-up data. A trial, on the other hand, is its own ground truth for the population it enrolled; the open question is not whether the trial's internal findings are accurate, but whether those findings hold up outside the specific population, setting, or time horizon studied. In short, validating a model asks whether it is a faithful representation of reality, while validating a trial-based analysis asks whether its results travel to other contexts. This distinction should be stated explicitly in the protocol, since it is the conceptual justification for why TBEE validation needs its own framework rather than simply adopting the MBEE taxonomy.

I am detailing other minor comments below for the authors' review:

Since this scoping review is on economic evaluations, I am not sure why any database that is specific to economics, such as EconLit, was not chosen.

Page 5, Eligibility Criteria: The authors state: "We will include studies conducted in any health condition, patient population, or setting, with no restrictions by disease area, population group, country, or healthcare system." However, in Table S4 (Data Extraction Form), specific diseases (such as Cancer, Psychiatry, Respiratory) and countries (UK, US, SE, NL, CA) are reported without rationalizing the selection. In Table 2 Search Strategy), the currencies reported (row 13 for Embase and row 15 in Medline) are also tied to specific countries, which needs further explanation. Also, rows 51 and 52 for Embase database search include rats etc., and animal experiments, though the protocol focuses on patient populations!

Table S1: The Registration row mentions PROSPERO. Note that does not accept scoping review protocols (it requires synthesis of a health-related outcome). OSF (Open Science Framework) Registry is the standard alternative for scoping review protocols. I suggest using OSF Registry instead, citing JBI methodology as the basis.

Optional but recommended to pre-specify:

- Whether stakeholders will be consulted (JBI-recommended optional stage)
- Whether funding sources of included studies will be reported

Limitation: An anticipated limitation involves heterogeneous and inconsistent terminology across the literature that may complicate systematic categorization.

VERSION 1 - AUTHOR RESPONSE

REVIEWER 1

A very timely, useful and welcome SR on trial-based economic evaluations.

Response: Thank you.

REVIEWER 2

I have a major concern about the statement that this scoping review will be conducted in accordance with the PRISMA-ScR guidelines. PRISMA-ScR is a reporting guideline for the final published scoping review, not technically a protocol template. There is no PRISMA-P equivalent specifically for scoping review template. For the protocol stage, the field instead leans on: 1) PRISMA-ScR checklist, applied prospectively — most protocols map their planned methods section onto the PRISMAScR items in advance, so the eventual review naturally satisfies reporting requirements; and 2) JBI methodology for scoping reviews — the companion methodological guidance (distinct from the reporting checklist) that PRISMA-ScR itself was built alongside, covering how to actually design and conduct each step (Ref: Peters MDJ, Marnie C, Tricco AC, et al. Updated methodological guidance for the conduct of scoping reviews. JBI Evid Synth. 2020;18(10):2119-2126. doi:10.11124/JBIES-20-00167).

Response: We thank the reviewer for this comment. We agree that PRISMA-ScR is a reporting guideline rather than methodological guidance for conducting a scoping review. We have revised the manuscript to clarify that PRISMA-ScR will be used to report the completed review, rather than to describe the conduct of the review.

Revised text (Abstract - Methods and analysis):

> This protocol describes a planned scoping review. The completed review will be conducted in accordance with the PRISMA-ScR guidelines.

Revised text (Methods - Study design):

> This protocol describes a planned scoping review. The completed review will be reported in accordance with the Preferred Reporting Items for Systematic Reviews and Meta-Analyses extension for Scoping Reviews (PRISMA-ScR). In the absence of a dedicated reporting guideline for scoping review protocols, this protocol has been informed by the PRISMA-Protocols (PRISMA-P) guideline (Table S1).

Also, the third paragraph of the Introduction raises an important conceptual distinction worth preserving clearly in the text: model-based economic evaluations (MBEEs) rely on an established, named validation taxonomy — face validity, internal verification, cross-validation, and external/predictive validation — drawn from Eddy et al. (2012) and operationalized in tools like AdViSHE. Trial-based economic evaluations (TBEEs), by contrast, rarely use "validation" as a term at all. Instead, the literature describes related but distinct concerns using different vocabulary: internal validity, external validity/generalizability, and transferability. This difference exists because models and trials rest on different epistemic foundations. A model has no single ground truth of its own — it synthesizes data from multiple sources, so its credibility must be checked against external benchmarks such as registries, comparator models, or longer-term follow-up data. A trial, on the other hand, is its own ground truth for the population it enrolled; the open question is not whether the trial's internal findings are accurate, but whether those findings hold up outside the specific population, setting, or time horizon studied. In short, validating a model asks whether it is a faithful representation of reality, while validating a trial-based analysis asks whether its results travel to other contexts. This distinction should be stated explicitly in the protocol, since it is the conceptual justification for why TBEE validation needs its own framework rather than simply adopting the MBEE taxonomy.

Response: We thank the reviewer for this thoughtful observation. We agree that model-based and trial-based economic evaluations differ in important respects and that concepts such as external validity, generalisability and transferability are more commonly discussed in the trial-based literature than validation per se. However, validation in trial-based economic evaluation remains poorly conceptualised and may encompass a broader range of activities than those relating solely to transferability, including checks of data, analytical code, assumptions, internal consistency, and the interpretation of findings. As the purpose of this scoping review is to map how validation is conceptualised in the literature, we have revised the Introduction to clarify this

distinction while retaining a deliberately broad conceptualisation of validation, rather than defining it a priori.

Revised text (Introduction, paragraph 3):

> Although concepts such as internal validity, external validity, generalisability and transferability are frequently discussed in relation to trial-based economic evaluations, they are not typically brought together within an overarching framework of validation. Consequently, it remains unclear which activities should be considered validation in this context and how they relate to existing concepts used in trial-based research.

I am detailing other minor comments below for the authors' review:

Since this scoping review is on economic evaluations, I am not sure why any database that is specific to economics, such as EconLit, was not chosen.

Response: The search strategy was developed in consultation with an experienced information specialist to maximise retrieval of literature relevant to validation in trial-based economic evaluations. Our choice of databases was informed by evidence on the identification of economic evaluations, including Arber et al. (2018), who found that searching key biomedical and health technology assessment databases captured the vast majority of published economic evaluations and concluded that searching beyond these databases may be inefficient when sensitive search strategies are used. Accordingly, we searched MEDLINE, Embase, INAHTA, and extensive targeted grey literature sources, which we considered appropriate for the objectives of this scoping review. We have revised the manuscript to explain the choice of database.

Revised text (Methods – Information source, paragraph 1):

> The choice of databases was informed by published evidence on the identification of economic evaluations, which supports the use of key biomedical and health technology assessment databases.

Page 5, Eligibility Criteria: The authors state: "We will include studies conducted in any health condition, patient population, or setting, with no restrictions by disease area, population group, country, or healthcare system." However, in Table S4 (Data Extraction Form), specific diseases (such as Cancer, Psychiatry, Respiratory) and countries (UK, US, SE, NL, CA) are reported without rationalizing the selection.

Response: The disease areas and countries listed in Table S4 are not eligibility criteria but response options included within the draft data extraction form to facilitate consistent data extraction and minimise free-text entry. The presence of these options does not restrict inclusion. As stated in the eligibility criteria, studies conducted in any health condition, patient population, setting, country, or healthcare system are eligible.

Any disease area or country not represented by a predefined option would be recorded using the "Other (specify)" field.

In Table 2 Search Strategy), the currencies reported (row 13 for Embase and row 15 in Medline) are also tied to specific countries, which needs further explanation.

Response: The currency terms included in the search strategy were taken from the validated MEDLINE and Embase search filters for economic evaluations developed by Hubbard et al. (Reference 17). They are included to maximise retrieval of economic evaluation studies and are not intended to restrict the search to particular countries or currencies.

Also, rows 51 and 52 for Embase database search include rats etc., and animal experiments, though the protocol focuses on patient populations!

Response: Rows 51-54 of the Embase search strategy form part of the validated trial search filter and are used to identify and exclude animal-only studies from the search results. These terms do not broaden the search to include animal experiments; rather, they improve the specificity of the search for studies involving human participants.

Table S1: The Registration row mentions PROSPERO. Note that does not accept scoping review protocols (it requires synthesis of a health-related outcome). OSF (Open Science Framework) Registry is the standard alternative for scoping review protocols. I suggest using OSF Registry instead, citing JBI methodology as the basis.

Response: We thank the reviewer for this suggestion. We agree that the Open Science Framework (OSF) provides an appropriate platform for prospective registration of scoping review protocols. However, this protocol was not prospectively registered. As this protocol has been submitted to BMJ Open for peer-reviewed publication prior to completion of the review, we have reported the registration item as "N/A" in accordance with the PRISMA-P checklist.

Optional but recommended to pre-specify:

• Whether stakeholders will be consulted (JBI-recommended optional stage)

Response: Stakeholder involvement is embedded within the wider programme of work in which this scoping review sits, including through involvement of methodological stakeholders in structured discussions to help interpret the review findings. Patient and public involvement is also planned, as described in the protocol. We have therefore not planned a separate stakeholder consultation stage as part of the scoping review.

• Whether funding sources of included studies will be reported

Response: As the purpose of this scoping review is to map how validation is conceptualised and undertaken in trial-based economic evaluations, we did not

consider funding sources of included studies to be directly relevant to the review objectives and therefore did not include them as a data extraction item.

Limitation: An anticipated limitation involves heterogeneous and inconsistent terminology across the literature that may complicate systematic categorization.

Response: We agree that variation in terminology across the literature may present challenges when identifying and categorising validation activities. We have incorporated this into the limitations of the review.

Revised text (Article Summary - Strengths and limitations):

- Eligibility requires validation to be explicitly described in the abstract. Heterogeneous and inconsistent terminology across the literature may further contribute to the exclusion of studies where validation activities are undertaken but not foregrounded.
