# Supplementary material for: Validation in trial-based economic evaluations: a scoping review protocol
Source: BMJ Open. 2026 Sep 17;16(9):e119447. doi: 10.1136/bmjopen-2026-119447 (PMC13599820; doi:10.1136/bmjopen-2026-119447)
Supplement: Supplementary file 2 [file bmjopen-16-9-s001.docx]

**Validation in trial-based economic evaluations: a scoping review protocol**

**SUPPLEMENTARY MATERIAL**

**Table S1. PRISMA-P (Preferred Reporting Items for Systematic review and Meta-Analysis Protocols) 2015 checklist[13]**

| **Section and topic** | **Item No** | **Checklist item** | **Page #** |
| --- | --- | --- | --- |
| **ADMINISTRATIVE INFORMATION** | | |  |
| Title: |  |  |  |
| Identification | 1a | Identify the report as a protocol of a systematic review | 1 |
| Update | 1b | If the protocol is for an update of a previous systematic review, identify as such | N/A |
| Registration | 2 | If registered, provide the name of the registry (such as PROSPERO) and registration number | N/A |
| Authors: |  |  |  |
| Contact | 3a | Provide name, institutional affiliation, e-mail address of all protocol authors; provide physical mailing address of corresponding author | 1 |
| Contributions | 3b | Describe contributions of protocol authors and identify the guarantor of the review | 12 |
| Amendments | 4 | If the protocol represents an amendment of a previously completed or published protocol, identify as such and list changes; otherwise, state plan for documenting important protocol amendments | N/A |
| Support: |  |  |  |
| Sources | 5a | Indicate sources of financial or other support for the review | 12 |
| Sponsor | 5b | Provide name for the review funder and/or sponsor | 12 |
| Role of sponsor or funder | 5c | Describe roles of funder(s), sponsor(s), and/or institution(s), if any, in developing the protocol | 12 |
| **INTRODUCTION** | | |  |
| Rationale | 6 | Describe the rationale for the review in the context of what is already known | 4 |
| Objectives | 7 | Provide an explicit statement of the question(s) the review will address with reference to participants, interventions, comparators, and outcomes (PICO) | 4-5 |
| **METHODS** | | |  |
| Eligibility criteria | 8 | Specify the study characteristics (such as PICO, study design, setting, time frame) and report characteristics (such as years considered, language, publication status) to be used as criteria for eligibility for the review | 5-6 |
| Information sources | 9 | Describe all intended information sources (such as electronic databases, contact with study authors, trial registers or other grey literature sources) with planned dates of coverage | 7 |
| Search strategy | 10 | Present draft of search strategy to be used for at least one electronic database, including planned limits, such that it could be repeated | 7-8 |
| Study records: |  |  |  |
| Data management | 11a | Describe the mechanism(s) that will be used to manage records and data throughout the review | 8 |
| Selection process | 11b | State the process that will be used for selecting studies (such as two independent reviewers) through each phase of the review (that is, screening, eligibility and inclusion in meta-analysis) | 8 |
| Data collection process | 11c | Describe planned method of extracting data from reports (such as piloting forms, done independently, in duplicate), any processes for obtaining and confirming data from investigators | 8 |
| Data items | 12 | List and define all variables for which data will be sought (such as PICO items, funding sources), any pre-planned data assumptions and simplifications | 8 |
| Outcomes and prioritization | 13 | List and define all outcomes for which data will be sought, including prioritization of main and additional outcomes, with rationale | 8-9 |
| Risk of bias in individual studies | 14 | Describe anticipated methods for assessing risk of bias of individual studies, including whether this will be done at the outcome or study level, or both; state how this information will be used in data synthesis | 9 |
| Data synthesis | 15a | Describe criteria under which study data will be quantitatively synthesised | 9 |
|  | 15b | If data are appropriate for quantitative synthesis, describe planned summary measures, methods of handling data and methods of combining data from studies, including any planned exploration of consistency (such as I^2^, Kendall’s τ) | N/A |
|  | 15c | Describe any proposed additional analyses (such as sensitivity or subgroup analyses, meta-regression) | N/A |
|  | 15d | If quantitative synthesis is not appropriate, describe the type of summary planned | 9 |
| Meta-bias(es) | 16 | Specify any planned assessment of meta-bias(es) (such as publication bias across studies, selective reporting within studies) | N/A |
| Confidence in cumulative evidence | 17 | Describe how the strength of the body of evidence will be assessed (such as GRADE) | N/A |

**Table S2. Search strategy**

Embase (1974 to 13 August 2025)

| 1 | cost benefit analysis/ | 101354 |
| --- | --- | --- |
| 2 | quality adjusted life year/ | 41554 |
| 3 | cost*.ti. | 214882 |
| 4 | (cost* adj2 utilit*).tw. | 14868 |
| 5 | (cost* adj2 (effective* or assess* or evaluat* or analys* or model* or benefit* or threshold* or quality or expens* or saving* or reduc*)).tw. | 457529 |
| 6 | (economic* adj2 (evaluat* or assess* or analys* or model* or outcome* or benefit* or threshold* or expens* or saving* or reduc*)).tw. | 78518 |
| 7 | (qualit* adj2 adjust* adj2 life*).tw. | 31664 |
| 8 | QALY*.tw. | 31037 |
| 9 | (incremental* adj2 cost*).tw. | 33083 |
| 10 | ICER.tw. | 15444 |
| 11 | utilities.tw. | 16990 |
| 12 | markov*.tw. | 45453 |
| 13 | (dollar* or USD or cents or pound or pounds or GBP or sterling* or pence or euro or euros or yen or JPY).tw. | 82862 |
| 14 | ((utility or effective*) adj2 analys*).tw. | 44568 |
| 15 | (willing* adj2 pay*).tw. | 17484 |
| 16 | (Eq 5D* or EQ-5D*).tw. | 29649 |
| 17 | ((euroqol or euro-qol or euroquol or euro-quol or eurocol or euro-col) adj3 ("5" or five)).tw. | 6731 |
| 18 | (european* adj2 quality adj3 ("5" or five)).tw. | 1284 |
| 19 | or/1-18 | 779089 |
| 20 | exp randomized controlled trial/ | 1096263 |
| 21 | Controlled clinical trial/ | 459936 |
| 22 | random$.ti,ab. | 2473667 |
| 23 | randomization/ | 101159 |
| 24 | intermethod comparison/ | 315652 |
| 25 | placebo.ti,ab. | 461553 |
| 26 | (compare or compared or comparison).ti. | 686965 |
| 27 | ((evaluated or evaluate or evaluating or assessed or assess) and (compare or compared or comparing or comparison)).ab. | 3307683 |
| 28 | (open adj label).ti,ab. | 186312 |
| 29 | ((double or single or doubly or singly) adj (blind or blinded or blindly)).ti,ab. | 365577 |
| 30 | double blind procedure/ | 306911 |
| 31 | parallel group$1.ti,ab. | 52510 |
| 32 | (crossover or cross over).ti,ab. | 155543 |
| 33 | ((assign$ or match or matched or allocation) adj5 (alternate or group$1 or intervention$1 or patient$1 or subject$1 or participant$1)).ti,ab. | 507560 |
| 34 | (assigned or allocated).ti,ab. | 603220 |
| 35 | (controlled adj7 (study or design or trial)).ti,ab. | 629109 |
| 36 | (volunteer or volunteers).ti,ab. | 321428 |
| 37 | human experiment/ | 723730 |
| 38 | trial.ti. | 555945 |
| 39 | or/20-38 | 7454569 |
| 40 | (random$ adj sampl$ adj7 ("cross section$" or questionnaire$1 or survey$ or database$1)).ti,ab. not (comparative study/ or controlled study/ or randomi?ed controlled.ti,ab. or randomly assigned.ti,ab.) | 10635 |
| 41 | Cross-sectional study/ not (exp randomized controlled trial/ or controlled clinical study/ or controlled study/ or randomi?ed controlled.ti,ab. or control group$1.ti,ab.) | 459952 |
| 42 | (((case adj control$) and random$) not randomi?ed controlled).ti,ab. | 24360 |
| 43 | Systematic review.ti,ab. not (trial or study).ti. | 416103 |
| 44 | (nonrandom$ not random$).ti,ab. | 21243 |
| 45 | random field$.ti,ab. | 3202 |
| 46 | (random cluster adj3 sampl$).ti,ab. | 1787 |
| 47 | (review.ab. and review.pt.) not trial.ti. | 1313260 |
| 48 | we searched.ab. and (review.ti. or review.pt.) | 59239 |
| 49 | update review.ab. | 154 |
| 50 | (databases adj4 searched).ab. | 78081 |
| 51 | (rat or rats or mouse or mice or swine or porcine or murine or sheep or lambs or pigs or piglets or rabbit or rabbits or cat or cats or dog or dogs or cattle or bovine or monkey or monkeys or trout or marmoset$1).ti. and animal experiment/ | 1312020 |
| 52 | Animal experiment/ not (human experiment/ or human/) | 2770249 |
| 53 | or/40-52 | 4950428 |
| 54 | 39 not 53 | 6563039 |
| 55 | 19 and 54 | 204038 |
| 56 | biomedical technology assessment/ | 19692 |
| 57 | (health technology assessment or medical technology assessment or technology appraisal? or hta or htai).ti,ab,kf,jw. | 20252 |
| 58 | 56 or 57 | 32435 |
| 59 | 55 or 58 | 233438 |
| 60 | ((identif* or find* or understand* or defin*) adj3 error?).ti,ab,kf. | 16574 |
| 61 | (model* adj5 error?).ti,ab,kf. | 21875 |
| 62 | (validat* or calibrat* or verif*).ti,kf. | 246778 |
| 63 | ((validat* adj3 calibrat*) or (validat adj3 verif*) or (calibrat* adj3 verif*)).ti,ab,kf. | 8195 |
| 64 | ((validat* or validity or calibrat* or identif* or performance) adj3 model?).ti,ab,kf. | 210455 |
| 65 | ((internal or external or face or cross or technical) adj validity).ti,ab,kf. | 18909 |
| 66 | (AdVi SHE or AdViSHE).ti,ab,kf. | 16 |
| 67 | 60 or 61 or 62 or 63 or 64 or 65 or 66 | 487265 |
| 68 | 59 and 67 | 6747 |
| 69 | limit 68 to "clinical trial (clinicaltrials.gov)" | 335 |
| 70 | limit 68 to (conference abstract or conference paper or "conference review") | 2242 |
| 71 | limit 68 to "preprint (unpublished, non-peer reviewed)" | 97 |
| 72 | 68 not (69 or 70 or 71) | 4073 |

Medline (Ovid MEDLINE® Epub Ahead of Print, In-Process & Other Non-Indexed Citations, Ovid MEDLINE® Daily and Ovid MEDLINE®) (1946 to 13 Aug 2025)

| 1 | Cost-Benefit Analysis/ | 98537 |
| --- | --- | --- |
| 2 | Quality-Adjusted Life Years/ | 18194 |
| 3 | Markov Chains/ | 17332 |
| 4 | exp Models, Economic/ | 16941 |
| 5 | cost*.ti. | 159407 |
| 6 | (cost* adj2 utilit*).tw. | 8880 |
| 7 | (cost* adj2 (effective* or assess* or evaluat* or analys* or model* or benefit* or threshold* or quality or expens* or saving* or reduc*)).tw. | 327665 |
| 8 | (economic* adj2 (evaluat* or assess* or analys* or model* or outcome* or benefit* or threshold* or expens* or saving* or reduc*)).tw. | 55196 |
| 9 | (qualit* adj2 adjust* adj2 life*).tw. | 20659 |
| 10 | QALY*.tw. | 16838 |
| 11 | (incremental* adj2 cost*).tw. | 20113 |
| 12 | ICER.tw. | 7399 |
| 13 | utilities.tw. | 10638 |
| 14 | markov*.tw. | 36236 |
| 15 | (dollar* or USD or cents or pound or pounds or GBP or sterling* or pence or euro or euros or yen or JPY).tw. | 60130 |
| 16 | ((utility or effective*) adj2 analys*).tw. | 29197 |
| 17 | (willing* adj2 pay*).tw. | 11893 |
| 18 | (Eq 5D* or EQ-5D*).tw. | 15538 |
| 19 | ((euroqol or euro-qol or euroquol or euro-quol or eurocol or euro-col) adj3 ("5" or five)).tw. | 4935 |
| 20 | (european* adj2 quality adj3 ("5" or five)).tw. | 892 |
| 21 | or/1-20 | 575970 |
| 22 | exp randomized controlled trial/ | 645945 |
| 23 | controlled clinical trial.pt. | 95725 |
| 24 | randomized.ab. | 704229 |
| 25 | placebo.ab. | 261056 |
| 26 | (drug therapy or therapy).fs. | 4995030 |
| 27 | randomly.ab. | 466517 |
| 28 | trial.ab. | 769012 |
| 29 | groups.ab. | 2898020 |
| 30 | **or/22-29** | 8185597 |
| 31 | 21 and 30 | 167312 |
| 32 | Technology Assessment, Biomedical/ | 11682 |
| 33 | (health technology assessment or medical technology assessment or technology appraisal? or hta or htai).ti,ab,kf,jw. | 11331 |
| 34 | 32 or 33 | 19308 |
| 35 | 31 or 34 | 183504 |
| 36 | ((identif* or find* or understand* or defin*) adj3 error?).ti,ab,kf. | 10207 |
| 37 | (model* adj5 error?).ti,ab,kf. | 19133 |
| 38 | (validat* or calibrat* or verif*).ti,kf. | 181421 |
| 39 | ((validat* adj3 calibrat*) or (validat adj3 verif*) or (calibrat* adj3 verif*)).ti,ab,kf. | 6737 |
| 40 | ((validat* or validity or calibrat* or identif* or performance) adj3 model?).ti,ab,kf. | 165197 |
| 41 | ((internal or external or face or cross or technical) adj validity).ti,ab,kf. | 14263 |
| 42 | (AdVi SHE or AdViSHE).ti,ab,kf. | 10 |
| 43 | 36 or 37 or 38 or 39 or 40 or 41 or 42 | 369209 |
| 44 | 35 and 43 | 3402 |
| 45 | limit 44 to preprint | 7 |
| 46 | 44 not 45 | 3395 |

International Network of Agencies for Health Technology Assessment (INAHTA) database (1989 to 13 Aug 2025)

| (validation OR validity)[Title] AND (model*)[Title] | 9 |
| --- | --- |
| (validation OR validity)[title] AND (cost* OR economic*)[title] | 5 |
| (validation OR validity)[abs] AND (model*)[title] AND (cost* OR economic*)[abs] | 5 |
| error*[title] AND model*[title] | 1 |
| error*[abs] AND model*[ti] AND (cost* OR economic*)[abs] | 23 |
| error*[title] AND (cost* OR economic*)[title] | 3 |

**Table S3. List of websites searched on 17 September 2025**

| **Source** | **Link** | **Hits** |
| --- | --- | --- |
| NICE | https://www.nice.org.uk/guidance/published | 0 |
| NICE DSU TSD | https://sheffield.ac.uk/nice-dsu/tsds/full-list | 27 |
| NICE DSU Methods | https://sheffield.ac.uk/nice-dsu/methods-development/nice-methods-guide-updates | 13 |
| CDA-AMC | https://www.cda-amc.ca/methods-and-guidelines | 12 |
| PBAC | https://pbac.pbs.gov.au/ | 16 |
| MSAC | https://www.msac.gov.au | 2 |
| ICER | https://icer.org/explore-our-research/policy-papers/ | 0 |
| WHO guidelines | https://www.who.int/publications/i?publishingoffices=c09761c0-ab8e-4cfa-9744-99509c4d306b | 0 |
| EU HTA network | https://health.ec.europa.eu/health-technology-assessment/publications_en | 0 |
| EU HTA network | https://health.ec.europa.eu/health-technology-assessment/key-documents_en | 1 |
| ProQuest | https://www.proquest.com/pqdtglobal | 635 |
| ISPOR | https://www.ispor.org/heor-resources/good-practices/report-other-report-types/-in-category/index-types/ispor-good-practices-for-outcomes-research | 4 |

CDA-AMC: Canada’s Drug Agency, DSU: Decision Support Unit, EU HTA: European Union Health Technology Assessment, ICER: Institute for Clinical and Economic Review, ISPOR: International Society for Pharmacoeconomics and Outcomes Research, MSAC: Medical Services Advisory Committee, NICE: National Institute for Health and Care Excellence, PBAC: Pharmaceutical Benefits Advisory Committee, TSD: technical support document, WHO: World Health Organization

**Table S4. Data Extraction Form**

| **Data item** | **Subitem** | **Option** | **Notes** |
| --- | --- | --- | --- |
| Study type | - | Trial-based economic evaluation  Methodological paper on trial-based economic evaluation  Guidance/framework | Trial-based economic evaluation includes RCTs, cluster RCTs, feasibility/pilot trials and other prospective studies described by authors as trials, such as pragmatic or quasi-experimental trials. |
| Type of economic evaluation | - | Not applicable  CBA  CCA  CEA  CUA  CMA |  |
| Health area | - | None  Cancer  Psychiatry  Public health  Respiratory  Surgery  Other (specify) |  |
| Country of analysis | - | UK  US  SE  NL  CA  International task force  Other (specify) | Two-letter country code used. |
| Validation activities | Focus aspect | Internal validity  External validity  Face/content validity  Cross validity  Predictive validity  Other (specify) | Focus aspect is based on ISPOR-SMDM Modeling Good Research Practices Task Force-7.[1] |
|  | Aspect validated | Not applicable  Resource use  Cost  Outcome(s)  Other (specify) | Multiple options allowed. |
|  | Stage | Design & recruitment  Data collection  Analysis |  |
|  | Basis of validation | Not applicable  Expert judgement  External empirical data  Internal trial data  Logic/internal consistency  Technical/ structural checks  Theory/ conceptual framework | Multiple options allowed. |
|  | Data source | Not applicable  Expert  Literature  Publicly available data  Routine data  Trial data | This is only filled in if the data source is empirical. Routine data includes medical records, social security records.  Multiple options allowed. |
|  | Approach | - | Free text due to the wide variation of methods used. |
|  | Part of QA | No  Yes | QA is defined as a deliberate activity undertaken to check, verify, test, or challenge the correctness, credibility, or generalisability of the economic evaluation. |
|  | Role of individual(s) conducting the validation | Same individual who conducted the economic evaluation  Different individual within the same research team  External to the study team  Unclear  Not applicable |  |
| Usefulness | Clarity of validation concept | High  Moderate  Low | High = Explicit definition and criteria, Moderate = Implicit or partial (i.e. validation is acknowledged and discussed but not conceptualised, defined, or structured/ it does not operationalise validation or conduct validation activities), Low = Unclear |
| Reviewer notes | Notes | - | Use this space for any reflections or to copy in brief quotes or passages from the authors that help clarify how validation was described |

CA: Canada, CBA: cost-benefit analysis, CCA: cost-consequences analysis, CEA: cost-effectiveness analysis, CMA: cost-minimization analysis, CUA: cost-utility analysis, IN: India, ISPOR-SMDM: International Society for Pharmacoeconomics and Outcomes Research - Society for Medical Decision Making, NL: The Netherlands, QA: quality assurance, SE: Sweden, UK: United Kingdom, US: United States
